# Modified OXIS Classification of Interproximal Contacts of Primary Canines and Its Prevalence in Six-to-Nine-Year-Olds in the Delhi National Capital Region: A Cross-Sectional Study

**DOI:** 10.7759/cureus.71611

**Published:** 2024-10-16

**Authors:** Madhulika Srivastava, Gauri Kalra, Carrolene Langpoklakpam, Rashika Singhania, Arjun P Mane

**Affiliations:** 1 Department of Pediatric and Preventive Dentistry, Manav Rachna Dental College, Faridabad, IND; 2 Department of Mathematics, Manav Rachna International Institute of Research and Studies, Faridabad, IND

**Keywords:** cross-sectional, deciduous teeth, interproximal contact, oxis, primary canine

## Abstract

Introduction: The modified OXIS (O - open contact, X - point contact, I - straight contact, S - curved contact) classification provides a useful framework for assessing interproximal contacts in primary canines and predicting caries risk. The study aimed to assess the incidence of modified OXIS classification of canine interproximal contacts in six-to-nine-year-old children from the Delhi National Capital Region (NCR) population.

Methods: A cross-sectional study was conducted to assess the interproximal contact areas between primary canine and first molar among six-to-nine-year-old children (n=400) according to the modified OXIS classification using the die stone study model. A single calibrated examiner performed a type III examination to assess a total of 1,536 contact areas from the occlusal aspect. The prevalence of each type was reported in terms of both numbers and percentages using the Chi-square test to find the prevalence, Fisher’s exact test to compare the inter- and intra-arch occurrence of the different contact types, and Kruskal-Wallis test to find its correlation with age.

Results: The Chi-square test showed that the most common contact type was I at 46.55% (715), followed by S1 at 28.06% (431), X at 18.56% (285), O at 6.19% (95), and S2 at 0.65% (10). The p-value of 0.000 indicated a statistically significant difference among the groups. The Kruskal-Wallis test found no statistically significant differences in patient ages. Additionally, Fisher’s exact test demonstrated a statistically significant prevalence of types O, I, and S1 when comparing them across arches.

Conclusion: Based on the current study, the most common contact type observed was type I, followed by type S1, according to the modified OXIS criteria. The findings indicate a higher prevalence of closed-type contacts in this population, suggesting that the occurrence of such contacts increases as permanent teeth erupt. This tool may be useful for assessing caries risk in this age group.

## Introduction

The interproximal contact area (ICA) is where two neighboring teeth connect. The areas above the ICA, known as spillway spaces or embrasures, provide two functions: they reduce stresses on the teeth by enabling food to escape from these contact areas while chewing and also prevent food lodgement between the teeth. The voids below these ICA, occupied by interdental papillae, are known as interproximal spaces [1].

The characteristics of these contact areas change depending on the type, alignment, and shape of the tooth. Existing literature indicates that primary molar contacts are generally broader, flatter, and positioned further toward the cervical area compared to permanent molar contacts [2]. Allison and Schwartz (2003) categorized contacts into two types: open and closed. These contacts can be either convex or concave, depending on their buccolingual or occlusal-cervical orientation [3].

Subramaniam et al. (2012) found that approximal caries are higher in the presence of closed contact in the primary dentition. This could be because of the tight contact between teeth that makes cleaning challenging, creating a major area for food lodgement [4]. Open and closed contacts are a rather more generalized classification and do not justify the diversity and complexity of primary tooth contact areas [5]. Consequently, many additional studies have been conducted to explore and classify the type of primary tooth contact areas [6]. Cortes et al. (2018) categorized these contacts as either convex or concave [7]. Similarly, Cho et al. (2021) categorized them into straight-straight, straight-concave, straight-convex, convex-convex, concave-concave, and concave-convex [2].

Muthu et al. (2018) introduced the OXIS (O - open contact, X - point contact, I - straight contact, S - curved contact) classification, a system that accounts for changes in the morphology of contact areas between primary molars [5]. However, this approach does not entirely address the anterior teeth. According to research, an anterior region in primary dentition typically has generalized spacing, is closed or crowded, and contains only primate spaces [8]. These findings imply that the existing classification system does not address the variations in the contact patterns of primary anterior teeth and canines [9].

To address this gap, Aarthi et al. (2022) conducted a study on children aged three to four years from the South Indian population. They modified the OXIS classification to account for changes in the interproximal contacts of primary canines. This new approach extended the classification from primary molars to include primary canines, as well as taking into account their rotation. According to this modification, the OXIS system was further divided into types O, X, I, S1, and S2 [9]. Therefore, the current study was done to assess the prevalence of modified OXIS classification of canine in six-to-nine-year-old children from the Delhi National Capital Region (NCR) population to analyze potential correlations between the occurrence of specific interproximal contact types and development of dental caries.

## Materials and methods

Ethical considerations

This cross-sectional study was carried out in the Department of Pediatric and Preventive Dentistry after gaining approval from the Institutional Ethics Committee of Manav Rachna Dental College, Faculty of Dental Sciences, Manav Rachna International Institute of Research and Studies under the reference number MRIIRS/MRDC/SDS/IEC/2024/127.

Study population and sample size

Patients were selected from both the Outpatient Department of the Department of Pediatric and Preventive Dentistry, Institute, and students from various nearby primary educational institutes in the Delhi NCR population. Using the G*Power software version 3.1.9.7 (Heinrich-Heine-Universität Düsseldorf, Düsseldorf, Germany), a total sample size of 400 children was found to be sufficient for the power of 80%, the confidence interval of 95%, and the effect size of 0.17 [9]. The inclusion criteria consisted of healthy, caries-free children aged six to nine years who were cooperative during impression-taking and had written parental consent. The exclusion criteria comprised uncooperative children, medically compromised children, as well as those with developmental defects, hypoplastic teeth, mobile teeth, carious or restored teeth, and teeth with crowns.

Clinical examination and contact area evaluation

Written informed consent from the parents was taken, and the children were thoroughly screened using a sterilized mouth mirror and probe. Intraoral photographs as well as impressions of the upper as well as lower arches from the study participants were taken with silicone-rubber-based impression material on a sectional impression tray. The impressions were then poured with die stone to cast the study models.

A single calibrated examiner conducted a type III examination to assess the contact area between the primary canines and first molars from an occlusal view. Before the study, a pediatric dentist received extensive training over 15 days to assess the contact areas following the modified OXIS classification. This training included both theoretical lectures and practical sessions, where the examiner evaluated 20 pairs (20 children) of study models under expert supervision. After 15 days following the completion of the training, the examiner conducted clinical examinations and assessed the contact types in those same 20 children. The Cohen kappa value was calculated to be 0.89, indicating a high degree of agreement.

**Figure 1:**
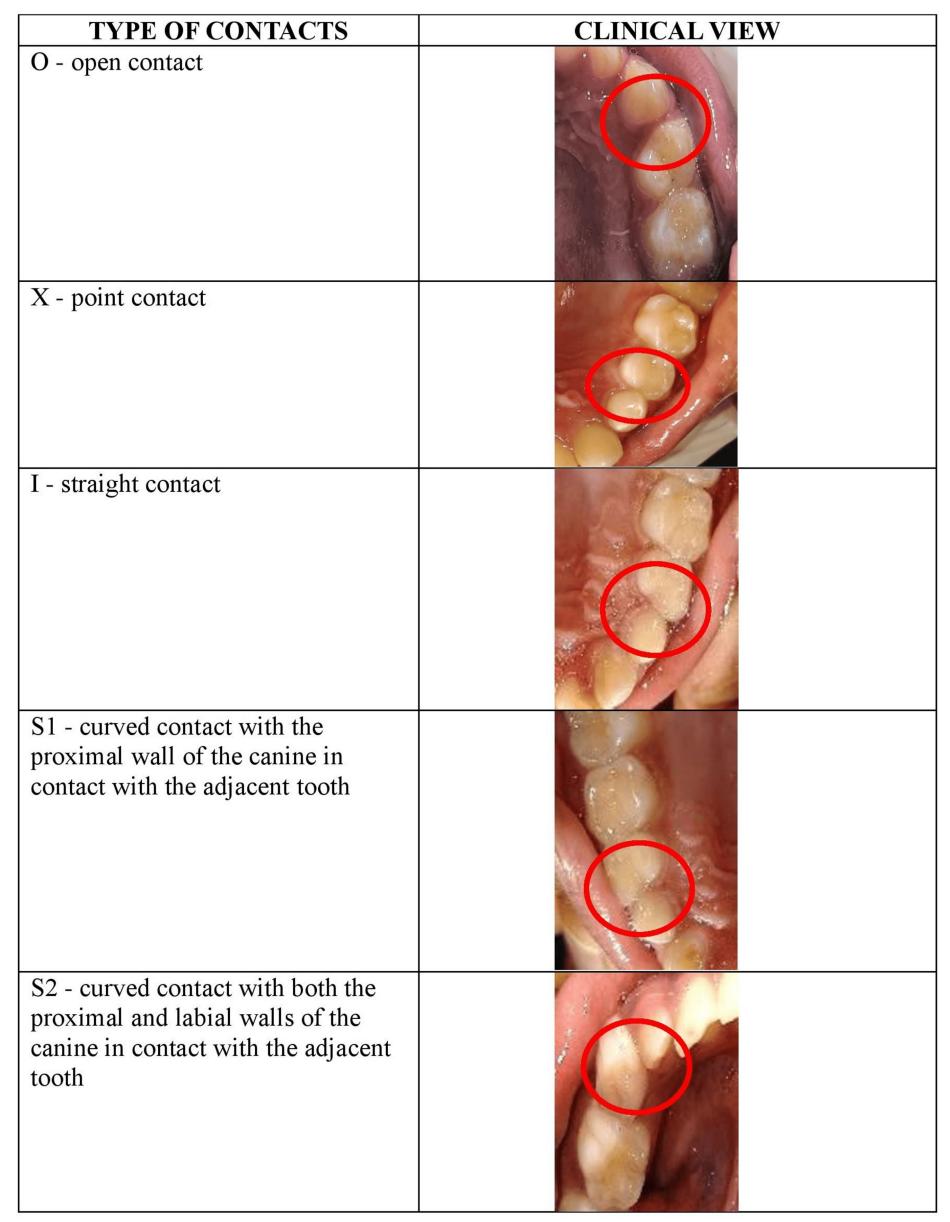
Illustrative images representing the type of contacts according to modified OXIS criteria

The contact areas between the canines and first molars in all four quadrants were evaluated, including both inter-arch and intra-arch contact areas. The contacts were categorized using the modified OXIS classification as follows: O (open contact), X (point contact), I (straight contact), S1 (curved contact with the distal wall of the canine in contact with the adjacent tooth), and S2 (curved contact with both the distal and labial walls of the canine in contact with the adjacent tooth), as depicted in Figure 1. The prevalence of each type was reported in terms of both numbers and percentages.

Statistical analysis

Statistical analysis was conducted with Microsoft Excel (Microsoft Corporation, Redmond, WA, USA) and Python version 3.11.4 (Python Software Foundation, Wilmington, DE, USA) under Pandas, NumPy, and Scipy libraries at a 95% confidence level. The prevalence across four types of the OXIS was analyzed for all four sides using the Chi-square test. The Kruskal-Wallis test was used to find the distribution of age across the type of OXIS with the Bonferroni correction method. The normality of the patient’s age was assessed using the Shapiro-Wilk test. Inter-arch and intra-arch relationships between the types of OXIS were analyzed using Fisher’s exact test.

## Results

The prevalence of various modified OXIS types across four measurement sites (upper and lower right and left) showed that type I is the most common, accounting for 46.55% (715) of the total. This was followed by type S1 at 28.06% (431), type X at 18.56% (285), type O at 6.19% (95), and type S2 at 0.65% (10). The p-value was 0.000, indicating a statistically significant difference (Table 1).

**Table 1:** Results of the Chi-square test determining the prevalence of types O, X, I, and S contacts * Statistically significant (significance level = 0.05) OXIS: O - open contact, X - point contact, I - straight contact, S - curved contact

Types of OXIS	Upper left (N=384)	Lower left (N=384)	Upper right (N=384)	Lower right (N=384)	OXIS prevalence (percentage %)	Chi-square p-value
O	18	19	38	20	95 (6.19%)	0.0000*
X	75	73	68	69	285 (18.56%)	
I	200	158	195	162	715 (46.55%)	
S1	88	131	81	131	431 (28.06%)	
S2	3	3	2	2	10 (0.65%)	

Based on the Kruskal-Wallis test, all p-values were above the adjusted significance level of 0.01, indicating statistically insignificant differences in patient ages among the modified OXIS types across the four measurements (Table 2).

**Table 2:** Age (in years) distribution of OXIS contact types across all four quadrants OXIS: O - open contact, X - point contact, I - straight contact, S - curved contact

Quadrants	O	X	I	S1	S2	Kruskal-Wallis (Bonferroni adjusted p-value)
Upper right	7.18 ± 1.83	7.78 ± 1.67	7.55 ± 1.67	8.01 ± 1.04	8.50 ± 0.71	0.6737
Upper left	7.39 ± 1.46	7.67 ± 1.73	7.51 ± 1.73	8.01 ± 1.03	8.33 ± 0.58	1
Lower right	7.60 ± 1.39	7.59 ± 1.36	7.75 ± 1.68	7.60 ± 1.61	7.00 ± 2.83	0.7391
Lower left	7.74 ± 1.41	7.56 ± 1.35	7.77 ± 1.68	7.60 ± 1.61	6.33 ± 2.52	0.4664

The Chi-square test results indicated no significant association between patient gender and the prevalence of modified OXIS types, as all p-values exceeded the 0.05 significance level (Table 3).

**Table 3:** Results of the Chi-square test to assess the correlation of OXIS contact types with gender OXIS: O - open contact, X - point contact, I - straight contact, S - curved contact

Quadrant	Gender	O	X	I	S1	S2	Chi-square statistic	p-value
Upper right	Male	16	35	97	42	2	3.1224	0.5375
	Female	22	33	98	39	0		
Upper left	Male	6	34	104	46	2	3.4885	0.4796
	Female	12	41	96	42	1		
Lower right	Male	10	34	86	61	1	1.2501	0.8698
	Female	10	35	76	70	1		
Lower left	Male	6	35	84	66	1	3.6761	0.4516
	Female	13	38	74	65	2		

The results for the different types of OXIS (O, X, I, S1, S2) contacts showed varying levels of significance when comparing the maxillary right vs. mandibular right and maxillary left vs. mandibular left. For types O and I, the odds ratios were significantly less than 1, indicating a lower likelihood of occurrence in the upper regions compared to the lower regions, with p-values of 0.0196 suggesting strong statistical significance. In contrast, no significant difference was seen with type X, as the odds ratio was close to 1 and p-values exceeded 0.05. However, type S1 showed odds ratios greater than 1, indicating a higher likelihood of occurrence in the upper regions compared to the lower regions, with very low p-values suggesting strong statistical significance. In contrast, type S2 had an odds ratio equal to 1, indicating no significant difference, and high p-values further support the lack of statistical significance (Table 4).

**Table 4:** Prevalence of OXIS contact types through inter-arch comparison OXIS: O - open contact, X - point contact, I - straight contact, S - curved contact

Types of OXIS	Inter-arch comparison			
	Maxillary right vs. mandibular right		Maxillary left vs. mandibular left	
	Odds ratio	Fisher's exact p-value	Odds ratio	Fisher's exact p-value
O	0.5003	0.0196	1.0585	1
X	1.0179	1	0.9671	0.9271
I	0.7073	0.0205	0.6432	0.003
S1	1.9369	0	1.7417	0.0008
S2	1	1	1	1

The intra-arch comparison for the different types of OXIS (O, X, I, S1, S2) reveals no significant differences in occurrence between the upper rights vs. upper left and lower right vs. lower left regions (Table 5).

**Table 5:** Prevalence of OXIS contact types through intra-arch comparison OXIS: O - open contact, X - point contact, I - straight contact, S - curved contact

Types of OXIS	Intra-arch comparison			
	Upper right vs. upper left		Lower right vs. lower left	
	Odds ratio	Fisher's exact p-value	Odds ratio	Fisher's exact p-value
O	0.4479	0.051	0.9473	1
X	1.1279	0.5782	1.0716	0.7804
I	1.0535	0.7728	0.9581	0.8262
S1	1.1121	0.6013	1	1
S2	1.5039	1	1.5039	1

## Discussion

This present study was done following the modified OXIS classification given by Aarthi et al. (2022) [9], being the first of its kind, evaluating the applicability of the classification among the North Indian population. Out of the 1,600 surfaces examined from 400 children, 1,536 interproximal contacts were assessed based on the inclusion criteria. Type I was found to be the most common, making up 46.55% (715) of the total, followed by S1, X, O, and S2. These results were contrary to the study done by Aarthi et al. (2022), who found the most common contact type was O and S was the least [9]. This may be because of the different age groups considered in both studies. Our study was conducted in children aged six to nine years, while Aarthi et al. considered children aged three to four years for their study.

By age three, the primary dentition is fully established and remains in the dental arches until around age nine. However, at about age six, when the early mixed dentition phase starts with the onset of the first permanent molar (FPM) eruption, several dynamic biological and functional changes occur [10]. The alveolar process undergoes remodeling to accommodate the eruption of the succedaneous teeth and starts building up a dental occlusion relationship between the upper and lower teeth [11].

When the FPM fully erupts into the oral cavity, it triggers an early mesial shift, which helps close the primate diastemas, and a late mesial shift, which closes the Nance's leeway space. Additionally, when the permanent central incisors start erupting, they apply lateral forces to the primary lateral incisors and canines, helping to close any remaining gaps [10-13]. This may explain why our study observed a higher prevalence of the closed type of ICA, particularly type I, compared to the open type, O, occurring at only 6.19% (95). This was in accordance with the study by Muthu et al. (2024), who concluded that as age advances, type O (open type) gets converted to types I, X, and S (closed types) [14].

It is possible that among the 400 children examined in this study, most would have exhibited type O contact as the most common type at ages three to four, but this likely changed over time with the eruption of the FPM. This hypothesis aligns with findings from the 2024 study by Muthu et al. (2024) [14], which noted significant changes in the OXIS contact type as age advances, with type O being the most unstable and undergoing the greatest transformation. This helps explain the discrepancy between our results and those of Aarthi et al. (2022) [9], who observed a higher prevalence of type O contact among three-to-four-year-olds.

When comparing contact types across the arch, types O, I, and S1 showed significant differences in their occurrence between the upper and lower regions, while types X and S2 did not. Since the Nance leeway space is greater in the mandible than in the maxilla, the FPM shifts its sagittal position relative to the maxillary FPM during the late mesial shift [15]. This shift may explain why type O was more common in the mandible, whereas the maxilla exhibited more of types I and S1. Additionally, in the mandibular arch, primate spaces are present distal to the primary canine [12,13,15] which could contribute to the higher prevalence of open-type contacts in the mandible, as the current study only considered distal contact of the canine.

When the comparison of various OXIS types was done within the arches, no significant difference was seen. There was no observed correlation between patient gender or age and the prevalence of different OXIS types. Due to a lack of existing literature on this topic, these findings cannot be compared with other studies, highlighting the need for further research.

The high prevalence of closed contacts, such as types I and S1, observed in this study is clinically significant because these types are particularly prone to caries, as noted in previous studies [5,16-18]. The close contact areas facilitate increased plaque deposition and food accumulation, making them difficult to clean and thus more susceptible to dental caries [6,9]. Understanding the type of contact between these teeth can serve as a valuable tool for caries risk assessment, aiding in the early prevention of caries development [14].

The current study had both strengths and limitations that are important to consider when evaluating its contributions to dental research. It was pioneering in examining the prevalence of the modified OXIS classification for interproximal contacts of canines in children aged six to nine within the Delhi NCR population. Understanding the different types of interproximal contacts during the mixed dentition stage is essential for several reasons, as this transition stage brings about numerous significant changes in dental anatomy and alignment. As the teeth erupt, the nature of interproximal contact can affect oral hygiene practices and, consequently, the risk of dental caries.

Closed-type contacts, such as I and S, are particularly more susceptible to plaque accumulation due to their challenging access to effective cleaning with standard brushing techniques compared to types O and X. This difficulty can create favorable conditions for bacterial growth and plaque accumulation, increasing the likelihood of dental caries in these areas. Therefore, early intervention such as implementing preventive strategies, enhancing oral hygiene practices, and promoting better long-term oral health is vital for caries prevention in this age group.

However, the study's limitations include the inability to compare its findings with existing research due to a lack of literature on this topic. Additionally, the results cannot be considered definitive, as a bigger sample size would have yielded better, more robust data. Future research with larger samples and more diverse populations is needed to draw more conclusive insights.

## Conclusions

Based on the current study, the most common contact type observed was type I, followed by type S1, according to the modified OXIS criteria. The findings indicate a higher prevalence of closed-type contacts in this population, suggesting that the occurrence of such contacts increases as permanent teeth erupt. This tool may be useful for assessing caries risk in this age group. However, to more accurately determine the prevalence and susceptibility to caries, further research involving larger and more diverse populations is needed.
